# *Mycoplasma pneumoniae* CARDS toxin elicits a functional IgE response in Balb/c mice

**DOI:** 10.1371/journal.pone.0172447

**Published:** 2017-02-15

**Authors:** Jorge L. Medina, Edward G. Brooks, Adriana Chaparro, Peter H. Dube

**Affiliations:** 1 Department of Microbiology and Immunology, University of Texas Health Science Center at San Antonio, San Antonio, Texas, United States of America; 2 Department of Pediatrics, Division of Immunology and Infectious Diseases, University of Texas Health Science Center at San Antonio, San Antonio, Texas, United States of America; Miami University, UNITED STATES

## Abstract

*Mycoplasma pneumoniae* is strongly associated with new onset asthma and asthma exacerbations. Until recently, the molecular mechanisms utilized by *M*. *pneumoniae* to influence asthma symptoms were unknown. However, we recently reported that an ADP-ribosylating and vacuolating toxin called the Community Acquired Respiratory Distress Syndrome toxin, CARDS toxin, produced by *M*. *pneumoniae* was sufficient to promote allergic inflammation and asthma-like disease in mice. A mouse model of CARDS toxin exposure was used to evaluate total and CARDS-toxin specific serum IgE responses. Mast cell sensitization, challenge, and degranulation studies determined functionality of the CARDS toxin-specific IgE. In the current study, we report that a single mucosal exposure to CARDS toxin was sufficient to increase total serum IgE and CARDS toxin-specific IgE in mice. Mice given a second mucosal challenge of CARDS toxin responded with significant increases in total and CARDS toxin-specific IgE. CARDS toxin-specific IgE bound to an N-terminal peptide of CARDS toxin but not the C-terminal peptide. Likewise, full-length CARDS toxin and the N-terminal peptide induced mast cell degranulation. Altogether, these data demonstrate that exposure to CARDS toxin is sufficient to generate functional IgE in mice. *M*. *pneumoniae* and CARDS toxin are strongly associated with asthma exacerbations raising the possibility that the CARDS toxin-specific IgE-mast cell axis contributes to disease pathogenesis.

## Introduction

Asthma and allergic diseases remain a significant source of morbidity and mortality in the developed world [1]. This is largely due to the complex interactions between the factors responsible for the etiology of asthma and allergic diseases [2]. Amongst the many factors contributing to allergic diseases; genetics, environment, the microbiota, and infectious agents have significant roles in pathogenesis [2–4]. There is strong clinical evidence that both viral and atypical bacterial agents are associated with worsening asthma, and there is growing experimental evidence that they play a role in the genesis of asthma[5–10].

Of the atypical pathogens, *Mycoplasma pneumoniae* is of particular interest due to prevalence in the community, the seasonal nature of infections, and the rapidly increasing rates of macrolide resistance in *M*. *pneumonia* [11–13] Currently *M*. *pneumoniae* is the leading cause of community acquired pneumonia amongst children in the US [11]. Depending on geographic location, macrolide resistance rates range form 95% in Asia to 10% in parts of Europe [12, 14]. *M*. *pneumoniae* colonizes tracheal and bronchial epithelium causing cytotoxicity characterized by loss of ciliary function and epithelial vacuolation [15, 16].

*Mycoplasma pneumoniae* has strong clinical associations with asthma exacerbations and morbidity in both children and adults [9, 13, 17]. Recently, a toxin produced by *M*. *pneumoniae*, the Community Acquired Respiratory Distress Syndrome toxin (CARDS toxin), was identified [18, 19]. CARDS toxin is an ADP-ribosylating and vacuolating cytotoxin that contributes to many of the pathologies observed during *M*. *pneumoniae* infection [10, 18, 20–24]. Recently we demonstrated that a single mucosal exposure to recombinant CARDS toxin is sufficient to induce an asthma-like pulmonary inflammation in naïve mice[10, 20], characterized by a dominant T-helper type-2 (Th2) response, peribronchiolar cellular inflammation, eosinophilia, mucus hypersecretion and goblet cell metaplasia[10, 20]. Additionally, these mice had increased airway resistance and decreased compliance following methacholine challenge. Altogether, these responses are characteristic of asthma-like inflammation.

*Mycoplasma pneumoniae* infection is strongly linked to exacerbations of asthma in children and adults[17, 25, 26]. We recently reported that children with refractory asthma and with CARDS toxin detected in their respiratory secretions reported a worsened quality of life and disease control relative to those that were CARDS toxin negative[13, 27], suggesting the toxin worsens disease.

Although many of the mechanisms leading to allergic inflammation remain poorly defined, the immunoglobulin-E (IgE) and mast cell axis are key mediators of the allergic reaction [28]. In animal models, an animal is typically exposed and becomes sensitized to an allergen only after multiple exposures, particularly if the exposures are mucosal (intranasal or intratracheal). Sensitized animals produce allergen-specific IgE that binds to high affinity IgE-receptors on basophils in the circulation and mast cells in the skin and mucosa [28, 29]. Sensitized basophils and mast cells can then respond almost instantaneously to a subsequent challenge with allergen resulting in rapid degranulation and mediator release [29]. Degranulation results in the immediate release of preformed effector molecules including proteases, biogenic amines, cytokines, and leukotrienes that mediate the physiological responses associated with allergy [30]. In addition to the pathologic role in allergy, antigen specific IgE has also been shown to have a protective role in honey bee and snake envenomation via degradation of toxin by mast cell-derived proteases [31, 32].

Classically, mast cells and IgE are considered protective against parasitic infections. We now appreciate that mast cells have a broader role in immunity providing protection against Gram-negative bacteria, *Mycoplasmas*, and viruses [33–39]. Likewise, there is precedence for bacterial toxins to enhance IgE-mediated responses, but this is a feature of the superantigen toxins from *Staphylococci* and *Streptococci* [40–42]. Interestingly, experimental vaccines using Pertussis toxin (PT), an ADP-ribosylating toxin, as an adjuvant leads to increases in IL-4 and IgE [43–45]. While it is well known that children with asthma are at higher risk for severe complications from pertussis [46], there is no evidence that pertussis is a significant factor in the genesis of asthma.

In this study we utilized our mouse model of CARDS toxin-associated asthma to test the hypothesis that exposure to CARDS toxin leads to the generation of a functional CARDS toxin-specific IgE.

## Materials and methods

### Animals

5-week-old BALB/cJ mice were purchased from Jackson Laboratory (Bar Harbor, ME) and STAT6-/- mice on the BALB/c background were provided by Dr. Michael Berton and bred in house. All mice were maintained in an AAALAC-approved facility with all animal work conducted according to relevant national and international guidelines. Work was conducted under a protocol approved by the University of Texas Health Science Center at San Antonio Institutional Animal Care and Use Committee (protocol #12070X) established. During experimental procedures, animals were monitored by laboratory staff twice a day and there were no apparent animal illnesses nor unexpected deaths during this study. Animal suffering was minimized by providing free access to food and water, animals had soft bedding for the duration of the experiments and all procedures were done under general anesthesia, 3% isoflurane and oxygen.

### Primary cells and cell lines

Bone marrow was collected from BALB/c mice and incubated in complete bone marrow mast cell (BMMC) media and incubated four weeks using a modified protocol previously described [47]. BMMC media contained RPMI 1640 medium, 10% fetal bovine serum, 20 ml 200 mM L-glutamine, 10 ml 10,000 U/ml penicillin/10,000 μg/ml streptomycin, 25 ml 1 M HEPES, 10 ml 100 mM sodium pyruvate, 10 ml 100× nonessential amino acids, 50 μM 2-mercaptoethanol (2-ME), 20 ng/ml murine IL-3 (Peprotech, Rocky Hill, NJ) and 20 ng/mL murine stem cell factor (SCF) (Peprotech). MC/9 cells were acquired from American Type Culture Collection (Manassas, VA) and cultured in BMMC media.

### Recombinant CARDS toxin

Recombinant CARDS toxin was kindly provided by Drs. Joel Baseman and TR Kannan. Briefly, rCARDS toxin was expressed and purified as previously described in detail [18, 20] and bioactivity assessed by its ability to induce vacuoles in HeLa cells [18, 20]. The rCARDS toxin vehicle (filter sterilized 50 mM tris buffer with 5% glycerol at pH 7.3) was used as a control. Recombinant CARDS toxin defective in ADP-ribosylation, CARDS E132A, was kindly provided by Dr. John Hart. Endotoxin concentrations in recombinant toxin were measured using Limulus Amebocyte lysate assays (Lonza, Walkersville, MD). Endotoxin was determined to be less than <0.1 EU/ml in all preparations used in this study.

### Exposure of animals to CARDS toxin

Mice were sedated with 3% isoflurane in air supplemented with oxygen. Seven hundred pmol of rCARDS toxin was administered IT via forced oropharyngeal aspiration as we have described [24]. The dose of toxin used in these studies was determined based on an analysis of CARDS toxin-mediated pathology in mice as previously described [20]. The actual concentration of CARDS toxin in the airways of humans during an asthma exacerbation is unknown. However, the doses used in this study are consistent with the levels detected in the epithelial lining fluid of baboons given a low-dose experimental infection of *M*. *pneumoniae*.

### Serum collection

Serum was allowed to clot at room temperature and stored overnight at 4°C. Samples were then centrifuged at 14000 rpm for 15 minutes at 4°C. Serum was collected and stored at -80°C.

### Total IgE and rCARDS toxin specific IgE assays

To measure total IgE, serum samples were diluted at 1:50 and assayed using a commercially available kit (BD Biosciences, San Jose, CA) according to the manufacturer’s instructions. rCARDS toxin-specific IgE was determined with a modified ELISA protocol previously described [48]. Briefly, ELISA plates were coated with 5 μg/mL α-mouse IgE antibody that was cross adsorbed to mouse IgM and IgG in carbonate buffer pH 9.5 and incubated overnight at 4°C. Plates were washed 3x with 0.05% PBS tween-20 (wash buffer) and blocked with 10% FBS in PBS (reagent buffer). Plates were washed as before with wash buffer and incubated with serum samples diluted in reagent buffer for 2 hours at room temperature. Plates were washed as before with wash buffer and incubated with 2.5 μg/mL rCARDS toxin in reagent buffer for 2 hours at room temperature. Plates were washed as before and incubated with antigen affinity purified rabbit anti-CARDS toxin IgG in reagent buffer for 1 hour at room temperature. Plates were washed 5x with wash buffer and incubated with goat anti-rabbit -IgG-HRP in reagent buffer for 1 hour at room temperature. Plates were washed 7x with wash buffer and incubated with substrate for 15 minutes (K-Blue Aqueous TMB reagent, Neogen Corporation, Lansing, MI). Reactions were stopped with 1M H_3_PO_4_ stop solution. Data represents two independent assays measured in triplicate.

### Western blots

2 μg of full-length rCARDS toxin, an amino terminus fragment (amino acids 1–249), a carboxy terminus fragment (amino acids 261–591), and ovalbumin were run on a 10% SDS PAGE gel. Gels were transferred to PVDF membranes (BioRad, Hercules, CA) using a semi-dry transfer Owl apparatus (Thermo Fisher Scientific, Waltham, MA). Membranes were blocked with 5% milk in TBS with 0.1% tween-20. Membranes were probed with serum from rCARDS toxin treated mice overnight at 4°C. Membranes were washed with TBS + 0.1% tween-20 and incubated with anti-mouse IgE cross-adsorbed to IgM and IgG in 5% milk in TBS with 0.1% tween 20 at room temperature for 2 hours. Membranes were washed and probed with a chicken anti-goat-HRP for 1 hour at room temperature. Membranes were washed and developed with enhanced luminol reagent (Perkin Elmer LAS Inc., Boston, MA) using autoradiography.

### Mast cell degranulation assay

A modified version of a previously described protocol was used [49]. Briefly, mast cells were sensitized overnight in BMMC media with a 1:10 dilution of complete serum from rCARDS toxin or vehicle challenged mice at a cell density of 1x10^6^. Mouse serum was not depleted of non-IgE immunoglobulins prior to sensitization. Cells were washed in Tyrode’s buffer (137 mM NaCl, 5.6 mM glucose, 2.7 mM KCl, 0.5 mM NaH_2_PO_4,_ 1.4 mM CaCl_2_, 0.5 mM MgCl, 10 mM HEPES, 0.1% BSA; pH 7.3) and resuspended in Tyrode’s buffer containing 10% deuterium to a density of 50,000 cell/well in a 96 well plate. Cells were challenged with full-length rCARDS toxin, rCARDS toxin fragments or controls and incubated 45 minutes. Cells were centrifuged and supernatant added to *p*-nitrophenyl *N*-acetyl-β-D-glucosamide (PNAG) (Sigma, St. Louis, MO) (3.5 mg/ml) dissolved in citrated buffer (40 mM citric acid/20 mM Na_2_HPO_4_·7 H_2_O; pH 4.5) to measure β-hexosaminidase activity. The cell pellet was lysed in Tyrode’s buffer with 0.1% Triton-X to measure total degranulation and incubated with PNAG. Supernatant and cell lysates were incubated at 37°C for 90 minutes. Reactions were stopped with 400 mM glycine pH 10.7 and plates read at 405 nm with a 620 nm reference filter. Percent hexosaminidase release was calculated as OD of the supernatant/ (OD of the supernatant + OD of the lysed cellular pellet) X100.

### Statistics

Experiments were repeated a minimum of 2 times with 6–10 animals, depending on the specific protocol (except where noted). All results were expressed as the mean ± S.D. Statistical differences were determined using a two-way ANOVA with Bonferroni post-hoc test or two-tailed Student *t-*test using GraphPad Prism version 5.04 (GraphPad Software, San Diego CA).

## Results

### A single exposure to CARDS toxin leads to increased total serum IgE

**Fig 1:**
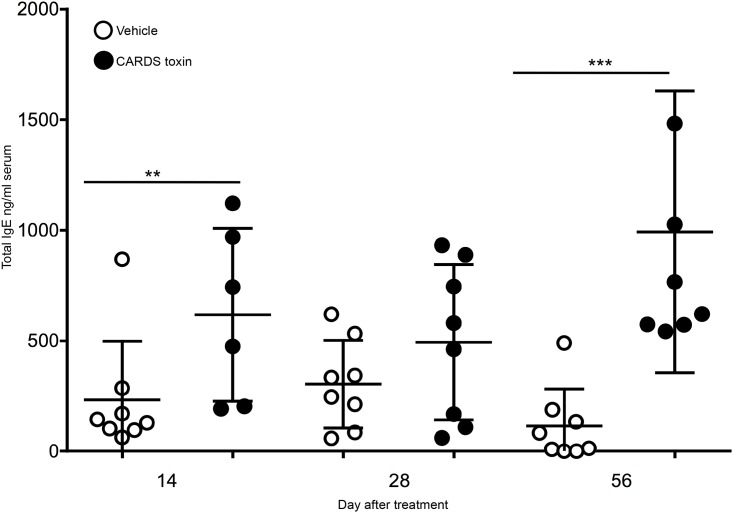
A single mucosal exposure leads to increased total serum IgE. Mice were treated IT with 700 pmol CARDS toxin or vehicle and then serum was collected at the indicated time points. Serum was diluted 1:50 and the concentration of IgE determined by ELISA. Data is presented as the mean and standard deviation of two independent experiments with each circle representing a mouse (** p<0.005, *** p<0.0005, N = 6–8 mice).

We previously reported that a single exposure to CARDS toxin results in prolonged changes in lung histopathology that lasted up to 56 days post-exposure [20]. Further, a single exposure to CARDS toxin was sufficient to cause asthma-like disease in toxin-naïve mice [10]. We hypothesized that the strong Th2 environment induced by CARDS toxin would promote production of IgE. As shown in Fig 1, Balb/cJ mice given a single intratracheal (IT) dose of CARDS toxin developed significant increases in total serum IgE ranging from 3 to 10-fold over vehicle controls on days 14 and 56 post-toxin exposure, respectively. While total serum IgE was increased on day 28 after toxin exposure relative to vehicle controls, these data were not statistically significant. Altogether, these data suggest that a single exposure to CARDS toxin is sufficient to induce increases in total serum IgE.

### Increases in serum IgE require CARDS toxin enzymatic activities and STAT6

**Fig 2:**
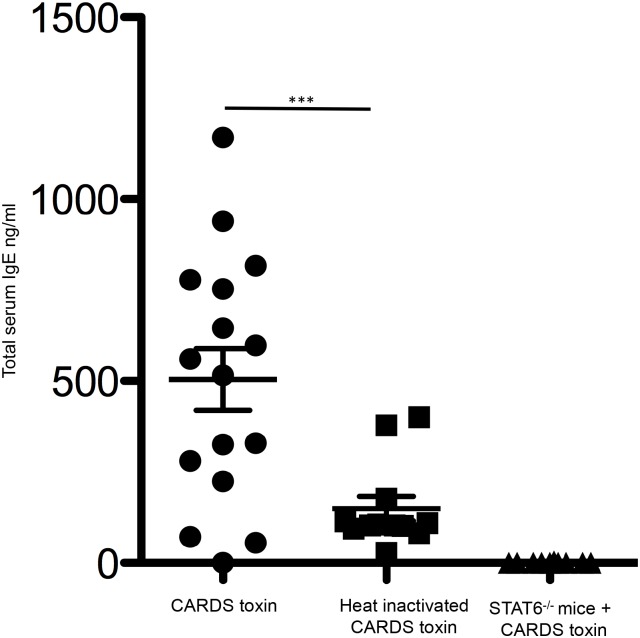
CARDS toxin activity and STAT6 are required for increased total serum IgE following CARDS toxin exposure. Balb/cJ mice or STAT6-/- mice were treated with active CARDS toxin or heat-inactivated CARDS toxin IT and then serum was collected after 56-days. Concentrations of total serum IgE is presented as the mean and standard deviation of two independent experiments. Each symbol represents a mouse N = 16 mice (***p< 0.0005 note that the STAT6-/- could not be evaluated statistically due to 15/16 values being undetectable).

Previously, we reported that heat-inactivated (HI) CARDS toxin does not induce the cytokine responses nor the characteristic changes in pulmonary histopathology observed with exposures to active toxin [20]. To test the role of CARDS toxin-activity and subsequent STAT6-dependent induction of serum IgE, Balb/cJ mice or STAT6-/- mice on the Balb/cJ genetic background were exposed IT to a single dose of CARDS toxin or HI CARDS toxin. Immunoglobulin class switching of antibodies to IgE requires IL-4 signaling through the IL-4R ultimately resulting in the activation of the STAT6 transcription factor [50]. As shown in Fig 2, STAT6 is required for CARDS toxin-mediated increases in total serum IgE. Further, mice exposed to HI CARDS toxin have, on average, 5-fold less total serum IgE than animals treated with active CARDS toxin (p< 0.0005). Importantly, these data with HI toxin suggest that the low levels of endotoxin present in recombinant toxin preparations (<0.1 EU/ml) are not sufficient to promote IgE production. Altogether, these data suggest that the enzymatic properties of CARDS toxin are required for increasing total serum IgE in a STAT6-dependent manner.

### Repeated exposures to CARDS toxin increases total serum IgE

**Fig 3:**
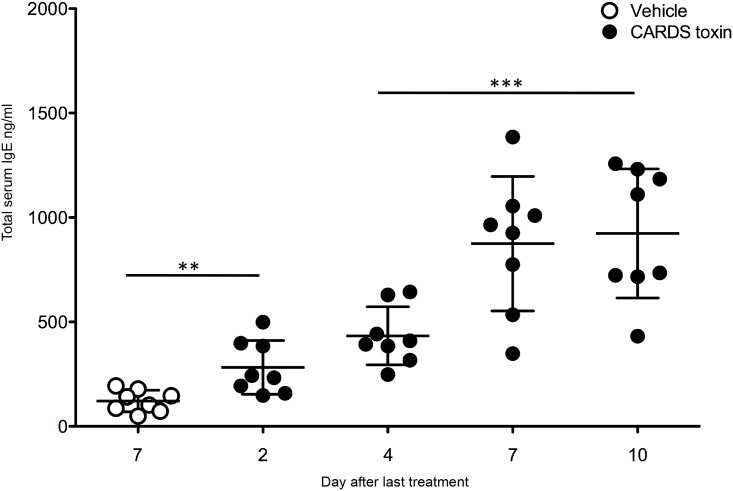
Multiple doses of CARDS toxin increase total serum IgE concentrations. Balb/cJ mice were treated IT with CARDS toxin or vehicle on day 0 and day 21. Mice were then bled on days 2, 4, 7, and 10 following the second exposure, and concentrations of total serum IgE measured. Total serum IgE from mice treated twice with vehicle were measured on day 7 after the second treatment. Data represents the mean and standard deviation of two independent experiments (**p<0.005, ***p<0.0005, N = 8 mice).

To test the ability of CARDS toxin to boost total serum IgE responses, mice were treated IT with CARDS toxin and then 21 days later mice received a second IT dose of CARDS TX. Total serum IgE concentrations were determined by ELISA on days 2, 4, 7, and 10 after treatment with the second dose of CARDS toxin. Following the second exposure to CARDS toxin, there was a rapid rise in total serum IgE evident by day 2 after-exposure that peaks at 10-fold over vehicle controls on days 7 and 10 after- exposure (p<0.005) (Fig 3).

### CARDS toxin exposure leads to the production of CARDS toxin-specific IgE

**Fig 4:**
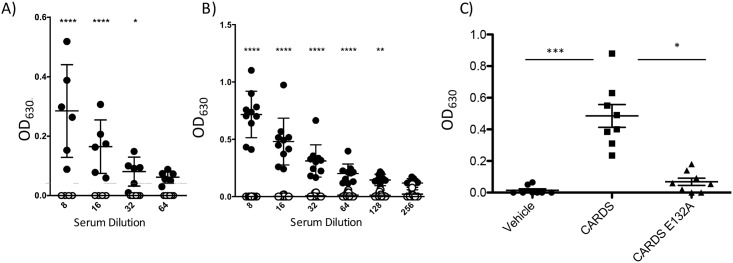
Exposure to active CARDS toxin results in CARDS toxin-specific IgE. **A)** Mice were exposed to a single dose of CARDS toxin IT and then serum collected on day 56. The indicated dilution of serum was tested for CARDS toxin-specific IgE in a sandwich ELISA. **B)** Mice were exposed to 700 pmol of CARDS toxin on days 0 and 21 IT and serum was collected on day seven after the second exposure. **C)** Mice were exposed to wild type or mutant CARDS toxin on days 0 and 21 and serum collected on day 28. (* p = 0.05, **p<0.005, ****p<0.0005 N = 6–10 mice/time point).

A single mucosal exposure of mice to CARDS toxin resulted in increased total serum IgE suggesting the production of CARDS toxin-specific IgE. As shown in Fig 4A, serum collected 56-days after a single IT exposure to CARDS toxin resulted in a significant increase in CARDS toxin-specific IgE concentrations measured by ELISA that remained significant when the serum was diluted to 1:32. Serum collected from days 14 and 28 also had significant increases in CARDS toxin-specific IgE (data not shown). Likewise, as shown in Fig 4B, when mice were exposed to 2 doses of CARDS toxin IT; the concentrations of CARDS toxin-specific IgE were significantly elevated and remained significant through the 1:128 dilution. Data in Fig 4B reflects serum harvested 7 days after the second exposure to CARDS toxin. Consistently, serum collected 2, 4, and 10 days after a second exposure to CARDS toxin had significant increases in CARDS toxin-specific IgE relative to controls (data not shown). To directly test the requirement for ADP-ribosylation activity in IgE production, Balb/cJ mice were treated IT with 700 pmol of CARDS toxin, CARDS toxin E132A, or vehicle. The E132A mutation disrupts the enzyme active site impairing ADP-ribosylation [51]. As shown in Fig 4C, concentrations of antigen-specific IgE were significantly reduced in the serum of animals treated with CARDS toxin E132A relative to active CARDS toxin at a 1:8 dilution. Altogether, these data indicate that mucosal exposure(s) to active CARDS toxin results in increased total and CARDS toxin-specific IgE.

### CARDS toxin-specific IgE recognizes epitopes in the N-terminus of CARDS toxin

**Fig 5:**
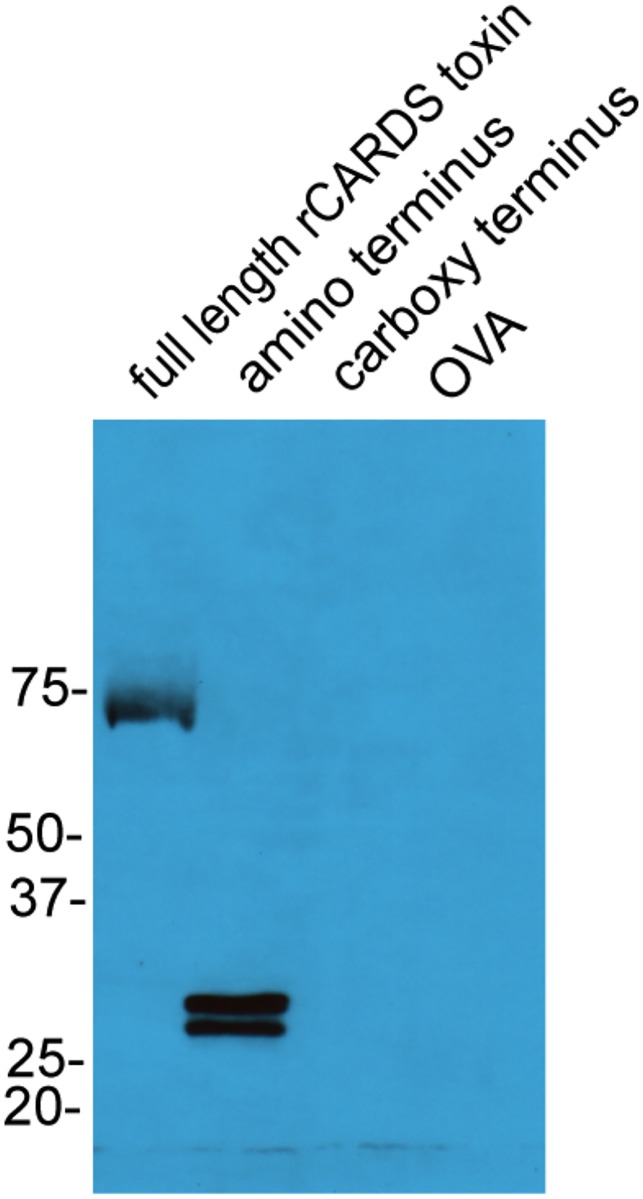
IgE from mice exposed to CARDS toxin recognizes full-length and N-terminal peptides of CARDS toxin on immunoblots. Serum collected from mice 56 days after exposure to CARDS toxin was pooled and used to probe immunoblots containing full-length CARDS toxin, the N-terminal 1–249 amino acid peptide, the amino acid 269–591 C-terminal peptide, and ovalbumin. Immunoreactive peptides were detected using an anti-mouse IgE antibody.

To partially define the region of CARDS toxin recognized by CARDS toxin-specific IgE, we tested immuno-reactivity in Western blots. Briefly, equimolar amounts of full length CARDS toxin, a 1–249 amino acid N-terminal fragment, a 261–591 amino acid C-terminal fragment, and chicken ovalbumin (negative control) were fractionated by gel electrophoresis and transferred to a PVDF membrane. The membrane was probed with pooled sera containing high titer anti-CARDS toxin-specific IgE. Immunoreactive bands were detected with an anti-mouse IgE secondary antibody and visualized with chemiluminescence. As shown in Fig 5., CARDS toxin-specific IgE recognizes full-length toxin and the N-terminal fragment but not a C-terminal fragment or ovalbumin. The 1–249 amino acid N-terminal fragment is present as two bands reflecting proteolytic cleavage of this peptide during purification. The N-terminal domain of the toxin contains the ADP-ribosylating activity [51]. These data suggest that IgE epitopes in CARDS toxin detectable by Western blot reside in the N-terminal 1–249 amino acids.

### Mast cells sensitized with CARDS toxin-specific IgE release hexosaminidase when challenged with CARDS toxin

**Fig 6:**
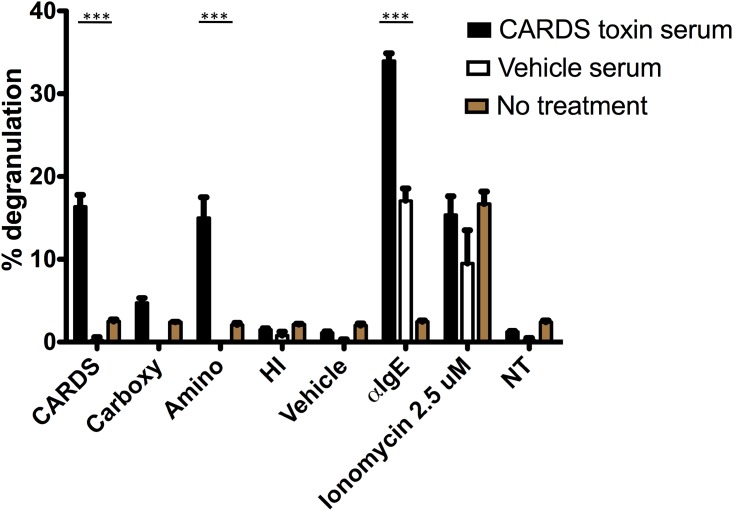
Mast cells sensitized with serum containing anti-CARDS toxin-specific IgE degranulate in response to CARDS toxin challenge. BMMCs were sensitized with pooled serum collected from mice 56 days after exposure to CARDS toxin or vehicle. As a negative control, BMMCs were mock sensitized with saline (NT). Sensitized mast cells were then challenged with equimolar amounts of full-length CARDS toxin, C-terminal peptide (amino acid 269–591), N-terminal peptide (amino acid 1–249), HI toxin, vehicle, anti-IgE, ionomycin, and mock challenged (NT). Percent degranulation is extrapolated from hexosaminidase release into the culture supernatant. (***p<0.005, data is presented as the mean and standard deviation of two independent experiments done in triplicate.)

To test if CARDS toxin-specific IgE was functional, mast cells were differentiated from mouse bone marrow (BMMC). BMMCs were then sensitized with pooled sera containing high titer anti-CARDS toxin IgE. As controls, BMMCs were sensitized with sera from vehicle control mice or untreated mice. Sensitized and non-sensitized mast cells were then challenged with different versions of CARDS toxin or nonspecific activators and hexoaminodase activity in the cell culture supernatants measured as an indicator of degranulation. Mast cells sensitized with serum containing anti-CARDS toxin-specific IgE and challenged with full length or the N-terminus of CARDS toxin resulted in 17–19% hexosaminidase release (Fig 6). Importantly, mast cells sensitized with CARDS toxin-specific IgE-containing serum did not respond to challenge with the C-terminal peptide of CARDS toxin. Degranulation of mast cells in response to challenge with CARDS toxin was specific since mast cells sensitized with sera from vehicle treated mice did not respond to challenge. Mast cells were capable of responding to nonspecific stimuli, ionomycin or anti-IgE treatment, with an 18% or 35% hexoaminidase release, respectively. Altogether, these data strongly suggest that a single mucosal exposure to CARDS toxin is sufficient to generate functional IgE in mice. Challenge with full length and N-terminal peptides of CARDS toxin induces mediator release in mast cells sensitized with anti-CARDS toxin IgE and demonstrates functionality in vitro.

## Discussion

Allergic diseases remain a significant source of morbidity and in the case of anaphylaxis, risk of mortality. Infection is a significant source of exacerbation in allergic asthma but the mechanisms underlying pathogen-associated disease worsening are poorly understood. Infections with *Mycoplasma pneumoniae*, respiratory syncytial virus, rhinovirus, and exposure to molds are known to enhance cytokine responses that promote allergic inflammation [5, 7–9, 13, 17, 52]. However, pathogen-associated virulence factors that influence allergic disease development and exacerbation are poorly understood.

We previously showed that CARDS toxin could induce allergic-like inflammation in BALB/c mice after a single challenge. This was defined by mucus hypersecretion, increased Th2 responses, eosinophilia, and airway hyperreactivity [10]. Additionally, we showed that rCARDS could exacerbate pre-existing allergic inflammation in BALB/cJ mice [24]. These data demonstrated that CARDS toxin could increase IL-4 and IL-13 mRNA expression, worsen airway eosinophilia and increase airway hyperreactivity in ovalbumin sensitized mice [24]. These aforementioned studies demonstrated increased type-2 inflammatory responses that could potentially affect IgE production, a central component of allergic inflammation. In the current study we evaluated the impact of CARDS toxin on IgE expression in BALB/cJ mice. Data presented shows that CARDS toxin elicits greater concentrations of total serum IgE in mice when mice are subjected to a mucosal CARDS toxin exposure(s). Additionally, we could detect CARDS toxin-specific IgE in the serum of mice given one or two doses of CARDS toxin, with the immunodominant epitope(s) located in the first 249 amino acids of the toxin. The N-terminal peptide was capable of degranulating sensitized mast cells and this peptide reacted with serum IgE from mice exposed to CARDS toxin on Western Blot. The increased immunoreactivity of the N-terminus could reflect differences in the immunogenicity of the N-terminus relative to other parts of the protein or it could have functional importance by directing antibodies to the portion of the protein with enzymatic activity. This could serve to directly neutralize the toxin or to indirectly neutralize it through the release of mast cell proteases. The biological and functional significance of toxin-specific IgE is currently under investigation. Finally, we detected mast cell degranulation due to cross linking of mast cell surface IgE with CARDS toxin, indicating that CARDS toxin-specific IgE is functional.

The C-terminal peptide of CARDS toxin did not react in immunoblots to sera containing CARDS toxin-specific IgE. Likewise, the C-terminal peptide of CARDS toxin did not result in mast cell degranulation when used to challenge sensitized mast cells. These results could imply that the majority of IgE epitopes are exclusively in the N-terminus of CARDS toxin. However, we cannot rule out a contribution of conformational or linear epitopes in the C-terminus that do not respond in our assays due to inappropriate protein folding. Further, we only used full length CARDS toxin to immunize animals and thus, did not test the ability of N- or C- terminal fragments to induce toxin-specific IgE directly. We did demonstrate that both heat inactivated toxin and the CARDS toxin E132A mutant do not induce a strong IgE response suggesting that the enzymatic activities of the toxin are required strengthening the suggestion that full-length toxin is required for optimal IgE responses.

*M*. *pneumoniae* infections are associated with increased total IgE, however it was unclear if CARDS toxin could replicate this aspect of *M*. *pneumoniae* infections [53]. IgE is the least abundant immunoglobulin class making up about 0.0005% of the total free serum immunoglobulins in non-atopic adults [54]. In atopic individuals, IgE concentrations are higher and can be a significant factor in allergic inflammation. For example, clinical studies report that elevated serum IgE is associated with worsened asthma symptoms and wheezing [54–56]. In this study, we demonstrate that both a single and double dose of CARDS toxin are sufficient to increase total serum IgE in BALB/c mice. Although not addressed in this study, the increased total IgE certainly reflects increases in CARDS toxin-specific IgE but could also represent IgE specific to other environmental allergens or self antigens. There is precedence in the literature supporting this concept in that a clinical study has shown shifts in IgE specific for other allergens in asthma patients with *M*. *pneumoniae* or RSV infections [53]. Likewise, emerging data suggests that in addition to its role in allergy and host defense, self-reactive IgE contributes to the pathology of systemic lupus erythematosus and atopic dermatitis [57–59]. Currently the reactivity of IgE to antigens other than CARDS toxin remains unknown in these animals.

Further, many environmental allergens associated with asthma exacerbations including house dust mites, cockroaches, fungi and many pollens [48] are known to exacerbate asthma symptoms in part due to elicited IgE responses [60–62]. Our data demonstrated that CARDS toxin enhances total IgE production in mice. This finding illustrates the potential of *M*. *pneumoniae* CARDS toxin to induce an IgE response and its potential to function as a classical allergen.

Mast cells are important to protect mice from *M*. *pneumoniae* infection [37]. However, in this previous work it was unclear if CARDS toxin-specific IgE was generated. It is now known, that aside from a role in protecting against helminth infection and mediating allergy, that mast cells are important cells of the innate immune system contributing to control of bacterial and viral infections [34–37, 39]. Likewise, IgE has been shown to have additional functions beyond mast cell and basophil activation especially in response to venoms [31, 32]. At this time, we have no data to support additional roles for CARDS toxin-specific IgE beyond mast cell sensitization but these other possibilities are currently being investigated.

Although many proteins have been identified as allergenic, bacterial toxins are not common allergens. *Staphylococcal* and *streptococcal* enterotoxins are potent inducers of IgE and toxin-specific IgE and represent the best-studied group of IgE-associated bacterial toxins [40–42]. Unlike CARDS toxin, these enterotoxins are superantigens with a very different mode of action and activity. CARDS toxin is an ADP-ribosylating and vacuolating toxin with the ADP-ribosylating activity residing in the N-terminus and the vacuolating activity in the C-terminus [22, 51]. There are reports of pertussis toxin inducing increased IgE responses when it is used as an experimental adjuvant for mucosal immunizations (38–40). Like CARDS toxin, pertussis toxin is an ADP-ribosylating toxin, but it lacks vacuolating activity. These data could suggest that ADP-ribosylating activity is required to generate toxin-specific IgE. We present data that mutant and heat-inactivated toxin fails to generate increased total IgE. Further, not all ADP-ribosylating toxins induce IgE responses. Diptheria toxin, a close relative of CARDS toxin, has been extensively studied and there is no evidence that it induces IgE responses. Cholera toxin (CT), another ADP-ribosylating toxin, has been used as an adjuvant for mucosal vaccines and one of the advantages of CT-based adjuvants is a lack of antigen-specific IgE production.

The mast cell-IgE axis is central to allergic inflammation. A series of signaling events occurs when mast cell surface bound IgE is cross-linked in the presence of allergen, leading to cytokine secretion, de novo synthesis of leukotrienes and mediator release/degranulation by mast cells [63]. These granules contain various inflammatory mediators such as histamine, serotonin and proteases, all of which have an impact on the allergic response [63]. Our studies reveal that CARDS toxin-specific IgE cross-linked in the presence of rCARDS toxin leads to mediator release. We cannot rule out a contribution from IgG immune complexes in our mast cell degranulation experiments since we did not deplete IgG from the serum prior to sensitization of the mast cells. However, IgG-immune complex-mediated degranulation of cells is a feature of anaphylaxis in mice. Recent reports suggest immune complexes might contribute to allergic inflammation [64]. In other studies, we evaluated animals for a drop in body temperature, an indicator of anaphylaxis in mice, following a second dose of CARDS toxin or vehicle and there were no significant differences between treatment groups (not shown). Altogether, these data support the IgE-mast cell axis in CARDS toxin-mediated allergic responses.

The presence of CARDS toxin-specific IgE in mice that can lead to IgE-dependent mediator release, suggests the possibility that CARDS toxin could be acting as an allergen in human asthma. While this remains to be determined, these findings add to rapidly accumulating evidence that CARDS toxin is a significant virulence factor with the potential to contribute to significant pathology in experimental and human asthma.
